# Risk factors and sequelae of epidermolysis bullosa acquisita: A propensity-matched global study in 1,344 patients

**DOI:** 10.3389/fimmu.2022.1103533

**Published:** 2023-01-26

**Authors:** Khalaf Kridin, Artem Vorobyev, Cristian Papara, David A. De Luca, Katja Bieber, Ralf J. Ludwig

**Affiliations:** ^1^ Lübeck Institute of Experimental Dermatology, University of Lübeck, Lübeck, Germany; ^2^ Azrieli Faculty of Medicine, Bar-Ilan University, Safed, Israel; ^3^ Unit of Dermatology and Skin Research Laboratory, Barch Padeh Medical Center, Tiberias, Israel; ^4^ Department of Dermatology, University Hospital Schleswig-Holstein Lübeck, Lübeck, Germany

**Keywords:** epidermolysis bullosa acquisita, TriNetX, risk factor, sequelae, systemic lupus erythematosus (SLE), inflammatory bowel disease, cardiovascular disease, lichen planus

## Abstract

Identification of risk factors and sequelae of any given disease is of key importance. For common diseases, primary prevention and disease management are based on this knowledge. For orphan diseases, identification of risk factors and sequelae has been challenging. With the advent of large databases, e.g., TriNetX, this can now be addressed. We used TriNetX to identify risk factors and sequelae of epidermolysis bullosa acquisita (EBA), a severe and orphan autoimmune disease. To date, there is only enigmatic information on EBA comorbidity. We recruited 1,344 EBA patients in the Global Collaborative Network of TriNetX. Using the “explore outcomes” function we identified 55 diagnoses with a different prevalence between EBA and no-EBA patients. We next performed propensity-matched, retrospective cohort studies in which we determined the risk of EBA development following any of the identified 55 diseases. Here, 31/55 diseases were identified as risk factors for subsequent EBA. Importantly, the highest risk for EBA were other chronic inflammatory diseases (CID), especially lupus erythematosus and lichen planus. Lastly, we determined the risk to develop any of the identified diseases after EBA diagnosis. Here, 38/55 diseases were identified as sequelae. Notably, EBA patients showed an increased risk for metabolic and cardiovascular disease, and thrombosis. Furthermore, the risk for CIDs, especially lupus erythematosus and lichen planus, was elevated. These insights into risk factors and sequelae of EBA are not only of clinical relevance, e.g., optimizing cardiovascular disease risk, but in addition, point to shared pathogenetic pathways between EBA and other inflammatory diseases.

## Introduction

Epidermolysis bullosa acquisita (EBA) is an orphan autoimmune disease that is characterized and caused by autoantibodies targeting type VII collagen (COL7). This short statement is grounded on research that was fronted by Detlef Zillikens: In 2005 his group demonstrated the induction of experimental EBA in mice following the transfer of antibodies targeting COL7 (1). At the same time, similar findings were published by the group of David Woodley (2). With these two landmark publications, the autoimmune pathogenesis of EBA had been firmly established. In parallel to this basic research on EBA, Detlef Zillikens also pursued epidemiological research questions, mainly aimed at the determination of the incidence and prevalence of autoimmune bullous dermatoses, such as EBA. Among other findings, he demonstrated a low EBA incidence and prevalence in Germany (3, 4).

Because EBA is such a rare disease, insights into risk factors and sequelae are sparse. Based on small cohorts, reviews of case reports and case report-series, an association between EBA and inflammatory bowel diseases (IBDs), such as ulcerative colitis (UC) and Crohn’s disease (CD), as well as infectious, cardiovascular, metabolic, malignant, and neurological diseases have been reported (5–12). Whilst no association of EBA with systemic lupus erythematosus (SLE) was observed, antinuclear antibodies (ANAs), a hallmark of SLE, were described to occur at higher frequencies than expected in patients diagnosed with EBA (12). Without exception, these reports are based on case reports, case report series or a meta-analysis of these. Thus, the evidence provided is of rather limited validity. In addition, all previous publications on that topic had reported associations only, with no information of the sequence of events. Hence, insights into the sequence of events are missing.

Understanding risk factors and sequelae of EBA would, however, significantly improve patient outcomes because risk factors could be addressed to prevent disease onset, and screening for diseases that subsequently develop after EBA diagnosis may contribute to their early detection. This is, for example, exemplified in the implementation of screening for metabolic, psychiatric, and cardiovascular comorbidity in psoriasis (13). In the same line, the European League Against Rheumatism (EULAR) recently published recommendations concerning lifestyle behavior to prevent progression of rheumatic diseases. In the context of non-inflammatory diseases, such as cardiovascular diseases, preventive measures to normalize elevated blood pressure or cholesterol are common clinical practice (14, 15). These include smoking cession and weight reduction in obese patients because nicotine dependence and overweight/obesity are known risk factors for the development of rheumatic diseases, being often associated with a more severe disease and a reduced response to treatment (16).

To define the so far enigmatic risk factors and associations of EBA, we assessed the Global Collaborative Network of TriNetX that provides access to anonymized, longitudinal patient data from close to 115 million individuals. First, we determined differences in disease prevalence in patients with EBA to those without. Next, we performed several retrospective cohort studies in which we determined the risk of subsequent EBA development following the diagnosis of any of the diseases found to be different in prevalence between EBA and no-EBA patients. Lastly, we determined the risk to develop any of the identified diseases after the diagnosis of EBA.

## Materials and methods

### Study design and database

**Figure 1:**
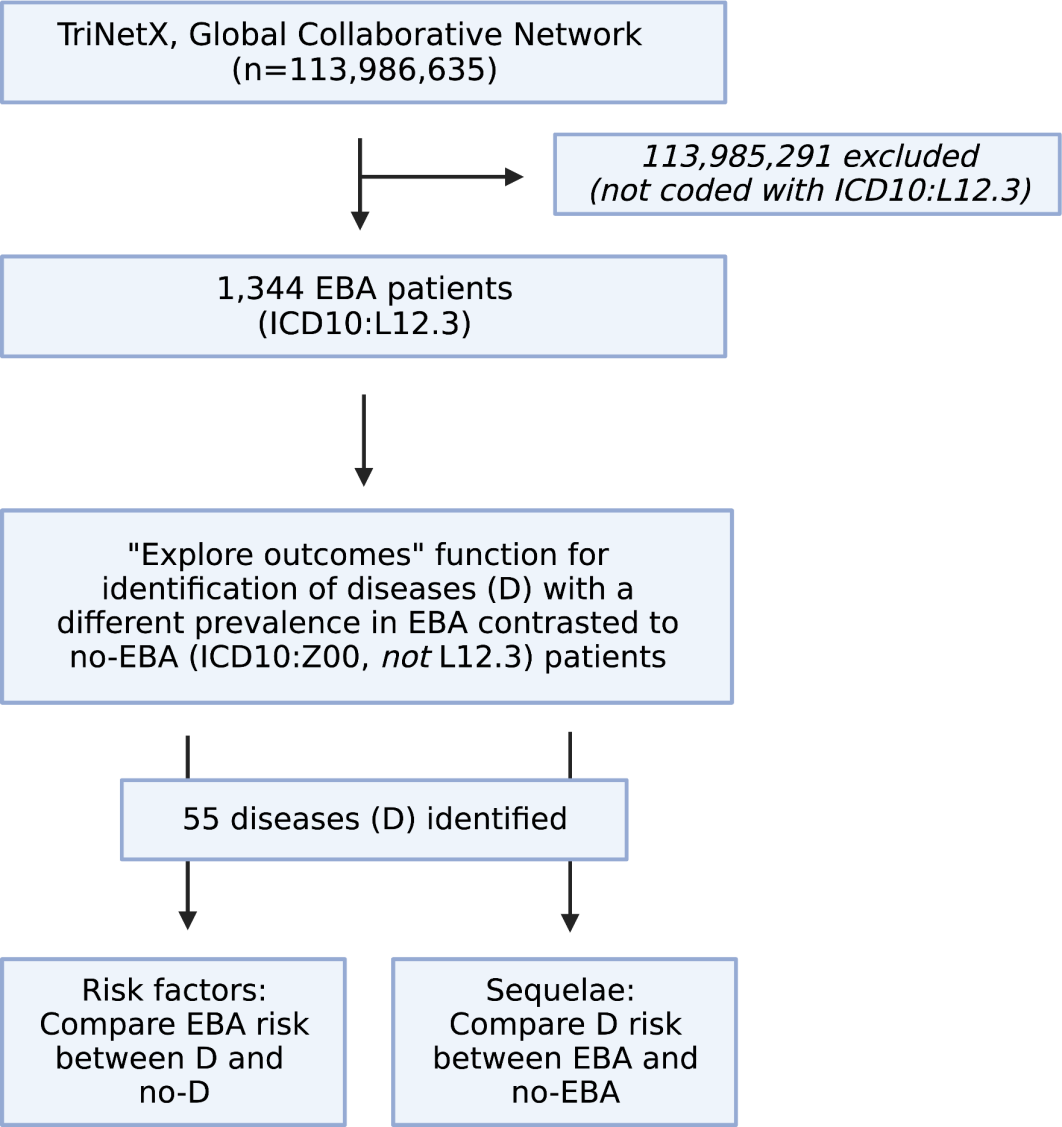
Study flow chart. Within the Global Collaborative Network of TriNetX that encompassed data from close to 115 million patients at the time of access (October 1^st^, 2022), 1,344 patients with the diagnosis of epidermolysis bullosa acquisita (EBA), defined by ICD10:L12.3 were identified. Patients without a diagnosis of EBA were excluded. Next, the “explore outcomes” function of TriNetX to identify diseases that are different in prevalence (at any given time) between EBA patients (defined by presence of ICD10:L12.3) those without EBA (defined as presence of ICD10:Z00 and absence of ICD10:L12.3). This identified 55 diseases with a different prevalence (at any given time) between EBA and no-EBA patients. Subsequently, 55 propensity-matched studies were performed to explore if any one of the identified diseases had an impact on subsequent EBA development. For this, patients with the disease, i.e., Crohn’s disease (ICD10:K50) were compared to those without the disease, i.g., no-Crohn’s disease (ICD10:Z00 and absence of ICD10:K50). Lastly, a propensity-matched study was performed to delineate the risk of EBA to develop any of the 55 identified diseases. Figure created with BioRender (https://biorender.com).

We performed a global population-based study with a propensity-matched retrospective cohort design. First, the Global Collaborative Network of TriNetX was used to compare differences regarding diagnoses of EBA patients (ICD10:L12.3) to those without EBA. For the latter group, individuals presenting for “Encounter for general examination without complaint, suspected or reported diagnosis” (ICD10CM:Z00) that had no diagnosis of EBA (ICD10CM:L12.3) were included. For the analysis, the “explore outcomes” function of TriNetX was used. Next, only diagnoses with a medium difference (2/3 bars) and a prevalence of 5% or more in EBA patients, and those with a high difference (3/3 bars) and a prevalence of 1% or more in EBA patients were considered further. We then performed the following retrospective cohort studies: First, the risk of EBA development following the diagnosis of any of the selected diseases was assessed. This was followed by the determination of development of any of the selected diseases following an EBA diagnosis (**Figure 1**).

### Study population and definition of eligible patients

The data used in this study was collected between October 1^st^ to 8^th^, 2022, from the TriNetX Global Collaborative Network, which, at the time of analysis, provided access to electronic medical records (diagnoses, procedures, medications, laboratory values, genomic information) from 113,986,635 million patients from 90 healthcare organizations (HCO). Propensity score matching was performed for the following variables: Age, sex, ethnicity, and race. TriNetX, LLC is compliant with the Health Insurance Portability and Accountability Act (HIPAA), the US federal law which protects the privacy and security of healthcare data, and any additional data privacy regulations applicable to the contributing HCO. TriNetX is certified to the ISO 27001:2013 standard and maintains an Information Security Management System (ISMS) to ensure the protection of the healthcare data it has access to and to meet the requirements of the HIPAA Security Rule, Any data displayed on the TriNetX Platform in aggregate form, or any patient level data provided in a data set generated by the TriNetX Platform, only contains de-identified data as per the de-identification standard defined in Section §164,514(a) of the HIPAA Privacy Rule. The process by which the data is de-identified is attested to through a formal determination by a qualified expert as defined in Section §164,514(b)(1) of the HIPAA Privacy Rule. Because this study used only de-identified patient records and did not involve the collection, use, or transmittal of individually identifiable data, this study was exempted from Institutional Review Board approval.

### Statistical analysis

Baseline characteristics were described by means and standard deviations (SDs) for continuous variables, and numbers and percentages for dichotomous variables. Continuous variables were compared using the student t-test, and dichotomous variables by Pearson chi-square test. Survival analyses were conducted by the Kaplan-Meier method. A log-rank test was run to determine if there were differences in the survival distribution for patients in the two investigated groups. Hazard ratios (HR)s for the study outcomes were obtained using the Cox regression model. Nelson-Aalen plots were utilized to test the proportional hazards assumption. Two-tailed P-values less than 0,05 were considered statistically significant.

## Results

### Cohort description and global results

**Figure 2:**
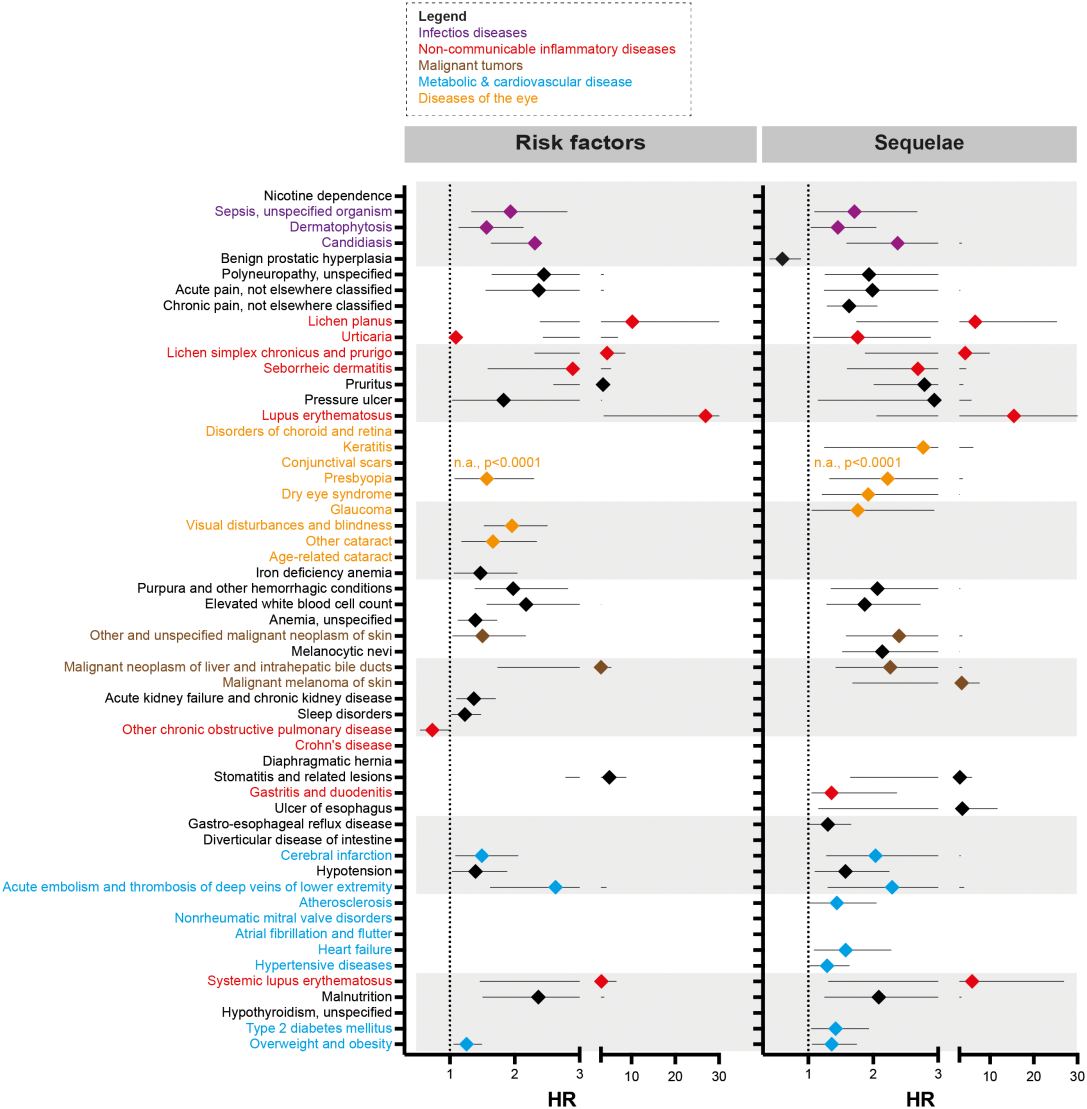
Risk factors and sequelae of epidermolysis bullosa acquisita. Hazard ratio (HR) event plots for the risk factors and sequelae of epidermolysis bullosa acquisita (EBA). Cohort descriptions and detailed results for risk factors are listed in **Supplement Tables 1–55** and **Table 1**, respectively. For risk factors, these are shown in **Supplement Table 56** and **Table 2**. Error bars indicate 5/95-percentiles. If no HR is shown, the indicated disease was not a risk factor / sequela of EBA.

For EBA-risk factors analysis, detailed cohort descriptions and results are displayed in **Supplement Tables 1–55** and **Table 1**. Sample sizes for the risk factor analysis ranged from 11,573 (conjunctival scars) to 7,849,544 (hypertensive diseases), with a median of 1,370,800 (845,473 to 2.337,172, 25/75-percentile). For each disease, propensity matching (age, sex, race, and ethnicity) was performed for better comparability of the groups. Globally, 31/55 diseases were associated with a higher risk of subsequent EBA, 23/55 with no impact on EBA risk, while 1/55 disease led to a reduced risk of subsequent EBA (**Figure 2** and **Table 1**). The corresponding data for the sequalae is found in **Supplement Table 56** and **Table 2**. Overall, we included 1,344 EBA patients (cases) and a similar number of controls. For a better comparability between cases and controls, they were again propensity matched for age, sex, race, and ethnicity. Between groups, no differences regarding these variables were observed. Overall, 37/55 diseases developed more frequently than expected following EBA diagnosis, for 17/55 diseases EBA was not a risk factor, and EBA had a lower risk for 1/55 diseases.

**Table 1:** Risk factors of epidermolysis bullosa acquisita (EBA).

		Cases			Controls					
Disease	ICD10 code	N of eligible participants	N of Outcomes	Risk, %	N of eligible participants*	N of Outcomes	Risk, %	Risk difference (95% confidence interval), %	Hazard ratio (95% confidence interval)	P value
Overweight and obesity	E66	6,923,027	280	0.004	6,923,063	245	0.004	0.001, 0-0.001	**1.257**, 1.059-1.493	0.0088
Type 2 diabetes mellitus	E11	5,637,675	240	0.004	5,637,665	255	0.005	–	–	n.s.
Hypothyroidism, unspecified	E03.9	3,402,569	145	0.004	3,402,556	133	0.004	–	–	n.s.
Malnutrition	E40-E46	919,769	46	0.005	919,811	33	0.004	0.001, 0-0.003	**2.363**, 1.505-3.71	0.0001
Systemic lupus erythematosus	M32	231,329	29	0.013	231,352	10	0.004	0.008, 0.003-0.014	**3.099**, 1.466-6.552	0.0018
Hypertensive diseases	I10-I16	7,840,452	284	0.004	7,840,405	307	0.004	–	–	n.s.
Heart failure	I50	2,460,148	84	0.003	2,460,164	113	0.005	–	–	n.s.
Atrial fibrillation and flutter	I48	2,440,224	84	0.003	2,440,208	113	0.005	–	–	n.s.
Nonrheumatic mitral valve disorders	I34	1,549,166	64	0.004	1,549,175	57	0.004	–	–	n.s.
Atherosclerosis	I70	1,246,293	57	0.005	1,246,328	50	0.004	–	–	n.s.
Acute embolism and thrombosis of deep veins of lower extremity	I82.4	845,427	53	0.006	845,451	24	0.003	0.003, 0.001-0.005	**2.628**, 1.622-4.259	< 0.0001
Hypotension	I95	1,837,108	89	0.005	1,837,163	84	0.005	0, -0.001-0.002	**1.395,** 1,035-1,882	0.0282
Cerebral infarction	I63	1,567,585	84	0.005	1,567,605	70	0.004	0.001, -0.001-0.002	**1,494**, 1,008-2,053	0.0126
Diverticular disease of intestine	K57	2,337,084	112	0.005	2,337,112	132	0.006	–	–	n.s.
Gastro-esophageal reflux disease	K21	6,973,564	295	0.004	6,973,597	251	0.004	–	–	n.s.
Ulcer of esophagus	K22.1	127,391	11	0.009	127,404	10	0.008	–	–	n.s.
Gastritis and duodenitis	K29	1,869,533	74	0.004	1,869,561	61	0.003	–	–	n.s.
Stomatitis and related lesions	K12	624,417	71	0.011	624,467	14	0.002	0.009, 0.006-0.012	**4.943**, 2.785-8.773	< 0.0001
Diaphragmatic hernia	K44	1,329,587	66	0.005	1,329,616	70	0.005	–	–	n.s.
Crohn’s disease	K50	294,555	20	0.007	294,562	11	0.004	–	–	n.s.
Other chronic obstructive pulmonary disease	J44	2,317,942	72	0.003	2,317,939	114	0.005	-0.002, -0.003-0.001	0.732 0.545-0.984	0.0378
Sleep disorders	G47	5,970,685	251	0.004	5,970,732	216	0.004	0.001, 0-0.001	**1.231,** 1.026-1.477	0.0250
Acute kidney failure and chronic kidney disease	N17-N19	3,557,269	169	0.005	3,557,299	158	0.004	0, -0.001-0.001	**1.371**, 1.103-1.704	0.0043
Malignant melanoma of skin	C43	282,444	24	0.008	282,476	13	0.005	–	–	n.s.
Malignant neoplasm of liver and intrahepatic bile ducts	C22	594,423	38	0.006	594,476	21	0.004	0.003, 0-0.005	**3.037**, 1.737-5.309	< 0.0001
Melanocytic nevi	D22	1,370,655	40	0.003	1,370,781	46	0.003	–	–	n.s.
Other and unspecified malignant neoplasm of skin	C44	1,080,602	73	0.007	1,080,674	47	0.004	0.002, 0-0.004	**1.504**, 1.042-2.17	0.0282
Anemia, unspecified	D64.9	4,097,465	190	0.005	4,097,521	148	0.004	0.001, 0-0.002	**1.394,** 1.124-1.728	0.0024
Elevated white blood cell count	D72.82	1,554,558	94	0.006	1,554,612	61	0.004	0.002, 0.001-0.004	**2.174**, 1.57-3.009	< 0.0001
Purpura and other hemorrhagic conditions	D69	1,325,255	79	0.006	1,325,309	50	0.004	0.002, 0.001-0.004	**1.977**, 1.387-2.82	0.0001
Iron deficiency anemia	D50	2,055,291	84	0.004	2,055,347	64	0.003	0.001, 0-0.002	**1.473**, 1.064-2.04	0.0190
Age-related cataract	H25	1,663,835	111	0.007	1,663,880	83	0.005	–	–	n.s.
Other cataract	H26	1,223,272	99	0.008	1,223,308	50	0.004	0.004, 0.002-0.006	**1.663**, 1.183-2.339	0.0031
Visual disturbances and blindness	H53-H54	3,457,195	179	0.005	3,457,247	98	0.003	0.002, 0.001-0.003	**1.956**, 1.529-2.503	< 0.0001
Glaucoma	H40-H42	1,221,974	77	0.006	1,221,991	47	0.004	–	–	n.s.
Dry eye syndrome	H04.12	908,659	57	0.006	908,695	35	0.004	–	–	n.s.
Presbyopia	H52.4	878,532	77	0.009	878,566	41	0.005	0.004, 0.002-0.007	**1.569**, 1.074-2.294	0.0190
Conjunctival scars	H11.2	11,556	14	0.121	11,573	0	0	0.121, 0.058-0.185%	**n.a.**	0.0001
Keratitis	H16	362,090	32	0.009	362,108	17	0.005	–	–	n.s.
Disorders of choroid and retina	H30-H36	1,476,209	82	0.006	1,476,231	61	0.004	–	–	n.s.
Lupus erythematosus	L93	92,859	28	0.03	92,904	10	0.011	0.019, 0.006-0.032	**26.957**, 3.666,198.223	< 0.0001
Pressure ulcer	L89	520,244	25	0.005	520,276	23	0.004	0, -0.002-0.003	**1.828,** 1.035-3.228	0.0348
Pruritus	L29	1,719,598	180	0.01	1,719,763	56	0.003	0.007, 0.005-0.009	**3.507**, 2.598-4.735	< 0.0001
Seborrheic dermatitis	L21	667,636	44	0.007	667,680	14	0.002	0.004, 0.002-0.007	**2.893**, 1.585-5.283	0.0003
Lichen simplex chronicus and prurigo	L28	257,992	48	0.019	258,031	11	0.004	0.014, 0.009-0.02	**4.446**, 2.309-8.561	< 0.0001
Urticaria	L50	1,072,233	69	0.006	1,072,291	18	0.002	0.005, 0.003- 0.006	**4.094**, 2.437-6.88	< 0.0001
Lichen planus	L43	74,580	21	0.028	74,609	10	0.013	0.015, 0-0.029	**10.198**, 2.391-43.499	< 0.0001
Chronic pain, not elsewhere classified	G89.2	4,199,569	152	0.004	4,199,663	169	0.004	–	–	n.s.
Acute pain, not elsewhere classified	G89.1	1,261,295	61	0.005	1,261,333	34	0.003	0.002, 0.001-0.004	**2.371**, 1.554-3.617	< 0.0001
Polyneuropathy, unspecified	G62.9	1,013,058	74	0.007	1,013,111	40	0.004	0.003, 0.001-0.005	**2.45**, 1.647-3.644	< 0.0001
Benign prostatic hyperplasia	N40	1,593,540	70	0.004	1,593,550	84	0.005	–	–	n.s.
Candidiasis	B37	1,933,646	107	0.006	1,933,718	46	0.002	0.003, 0.002-0.004	**2.311**, 1.636-3.266	< 0.0001
Dermatophytosis	B35	1,676,899	105	0.006	1,676,983	59	0.004	0.003, 0.001-0.004	**1.565**, 1.137-2.153	0.0056
Sepsis, unspecified organism	A41.9	1,336,969	62	0.005	1,337,011	52	0.004	0.001, -0.001-0.002	**1.935** 1.333-2.808	0.0004
Nicotine dependence	F17	5,515,751	196	0.004	5,515,748	213	0.004	–	–	n.s.

The risk of EBA development in individuals diagnosed with any one of the listed diseases cases to those without these diagnoses controls. Data shown displays results from measures of association excluding patients with EBA prior to the time window. For the Hazard ratio. Kaplan-Meier Analysis again excluding patients with outcome prior to the time window with Log-Rank Test was performed. N, number; n.s., not significant; n.a., not applicable; Diseases where presence of any of the diseases increases the risk are highlighted in bold. Diseases where presence of the indicated disease decreases the risk are highlighted in blue and bold. Diseases that do not impose a risk for subsequent EBA development are displayed in gray. Please note that propensity score matching is re-run with each outcomes analysis so that the analysis uses the most current data available on the TriNetX network. Analysis was performed from October 3rd to 8th, 2022.

**Table 2:** Sequelae of epidermolysis bullosa acquisita (EBA).

		Cases			Controls					
Disease	ICD10 code	N of eligible participants	N of Outcomes	Risk, %	N of eligible participants*	N of Outcomes	Risk, %	Risk difference (95% confidence interval), %	Hazard ratio (95% confidence interval)	P value
Overweight and obesity	E66	1,031	128	12.415	1,175	130	11.064	1.351, -1.345-4.047	**1.360**, 1.059-1.747	0.0156
Type 2 diabetes mellitus	E11	1,048	93	8.874	1,138	80	7.030	1.844, -0.430-4.118	**1.420**, (1.045-1.931	0.0245
Hypothyroidism, unspecified	E03.9	1,180	44	3.729	1,222	65	5.319	–	–	n.s.
Malnutrition	E40-E46	1,285	44	1,335	1,335	23	1.723	1.701, 0.486-2.916	**2.088**, 1.250-3.467	0.0036
Systemic lupus erythematosus	M32	1,301	11	0.846	1,338	10	0.747	0.098, -0.581-0.777	**5.940**, 1.311-26.908	0.0087
Hypertensive diseases	I10-I16	729	145	19.890	809	147	18.171	1.720, -2.212-5.65	**1.289**, 1.017-1.632	0.0350
Heart failure	I50	1,241	70	5.641	1,286	54	4.199	1.442, -0.246-3.130	**1.575**, 1.090-2.275	0.0147
Atrial fibrillation and flutter	I48	1,234	47	3.809	1,256	49	3.901	–	–	n.s.
Nonrheumatic mitral valve disorders	I34	1,275	40	3.137	1,304	51	3.911	–	–	n.s.
Atherosclerosis	I70	1,284	73	5.685	1,306	60	4.594	1.091, -0.610-2.792	**1.441**, 1.014-2.050	0.0407
Acute embolism and thrombosis of deep veins of lower extremity	I82.4	1,283	38	2.962	1,330	19	1.429	1.533, 0.407-2.659	**2.291**, 1.301-4.035	0.0032
Hypotension	I95	1,235	73	5.911	1,313	57	4.341	1.570, -0.1460-3.286	**1.574**, 1.102-2.249	0.0119
Cerebral infarction	I63	1,255	51	4.064	1,313	31	2.361	1.703, 0.336-3.069	**2.036**, 1.280-3.238	0.0022
Diverticular disease of intestine	K57	1,226	83	6.770	1,285	106	8.249	–	–	n.s.
Gastro-esophageal reflux disease	K21	1,023	137	13.392	1,150	139	12.087	1.305, -1.507-4.117	**1.303**, 1.024-1.659	0.0312
Ulcer of esophagus	K22.1	1,327	12	0.904	1,344	10	0.744	0.160, -0.526-0.846	**3.677**, 1.156-11.691	0.0195
Gastritis and duodenitis	K29	1,265	57	4.506	1,315	47	3.574	0.932, -0.589-2.453	**1.578**, 1.053-2.365	0.0259
Stomatitis and related lesions	K12	1,251	36	2.878	1,339	14	1.046	1.832, 0.757- 2.907	**3.108**, 1.648-5.862	0.0002
Diaphragmatic hernia	K44	1,274	56	4.396	1,311	46	3.509	–	–	n.s.
Crohn’s disease	K50	1,321	10	0.757	1,339	10	0.747	–	–	n.s.
Other chronic obstructive pulmonary disease	J44	1,261	49	3.886	1,287	52	4.040	–	–	n.s.
Sleep disorders	G47	1,069	124	11.6	1,193	126	10.562	–	–	n.s.
Acute kidney failure and chronic kidney disease	N17-N19	1,121	98	8.742	1,249	104	8.327	–	–	n.s.
Malignant melanoma of skin	C43	1,314	28	2.131	1,337	11	0.823	1.308, 0.389-2.227	**3.581**, 1.683-7.620	0.0004
Malignant neoplasm of liver and intrahepatic bile ducts	C22	1,311	54	4.119	1,338	32	2.392	1.727, 0.376- 3.079	**2.263**, (1.424-3.596	0.0004
Melanocytic nevi	D22	1,249	95	7.606	1,327	61	4.597	3.009, 1.157-4.862	**2.142**, 1.526-3.005	< 0.0001
Other and unspecified malignant neoplasm of skin	C44	1,244	69	5.547	1,308	40	3.058	2.489, 0.911-4.066	**2.398**, 1.582-3.636	< 0.0001
Anemia, unspecified	D64.9	1,126	119	10.568	1,261	129	10.23	–	–	n.s.
Elevated white blood cell count	D72.82	1,237	72	5.821	1,322	52	3.933	1.887, 0.214-3.561	**1.87**, 1.282-2.727	0.0010
Purpura and other hemorrhagic conditions	D69	1,246	61	4.896	1,310	36	2.748	2.148, 0.658-3.637	**2.064**, 1.348-3.16	0.0007
Iron deficiency anemia	D50	1,241	79	6.366	1,297	68	5.243	–	–	n.s.
Age-related cataract	H25	1,233	79	6.407	1,289	91	7.060	–	–	n.s.
Other cataract	H26	1,242	55	4.428	1,316	57	4.331	–	–	n.s.
Visual disturbances and blindness	H53-H54	1,151	85	7.385	1,270	90	7.087	–	–	n.s.
Glaucoma	H40-H42	1,266	38	3.002	1,303	30	2.302	0.699, -0.544-1.943	**1.762**, 1.057-2.940	0.0280
Dry eye syndrome	H04.12	1,286	49	3.81	1,325	34	2.566	1.244, -0.105-2.593	**1.923**, 1.213-3.05	0.0047
Presbyopia	H52.4	1,265	43	3.399	1,318	25	1.897	1.502, 0.262-2.743	**2.223**, 1.327-3.724	0.0019
Conjunctival scars	H11.2	1,327	13	0.98	1,344	0	0	0.98, 0.45-1.51	n.a.	0.0001
Keratitis	H16	1,311	22	1.678	1,339	10	0.747	0.931, 0.097-1.766	**2.77**, 1.247-6.156	0.0091
Disorders of choroid and retina	H30-H36	1,259	39	3.098	1,303	55	4.221	–	–	n.s.
Lupus erythematosus	L93	1,288	15	1.165	1,342	10	0.745	0.419, -0.326-1.164	**15.546**, 2.053-117.693	0.0004
Pressure ulcer	L89	1,310	32	2.443	1,336	13	0.973	1.470, 0.482-2.458	**2.942**, 1.513-5.723	0.0009
Pruritus	L29	1,111	112	10.081	1,330	61	4.586	5.495, 3.397-7.592	**2.791**, 2.010-3.877	< 0.0001
Seborrheic dermatitis	L21	1,269	49	3.861	1,334	20	1.499	2.362, 1.117-3.607	**2.688**, 1.597-4.525	0.0001
Lichen simplex chronicus and prurigo	L28	1,284	27	2.103	1,341	10	0.746	1.357, 0.447-2.267	**4.314**, 1.877-9.914	0.0002
Urticaria	L50	1,261	41	3.251	1,335	30	2.247	1.004, -0.257-2.260	**1.763**, 1.078-2.885	0.0222
Lichen planus	L43	1,308	15	1.147	1,342	10	0.745	0.402, -0.336-1.140	**6.640**, 1.744-25.278	0.0017
Chronic pain, not elsewhere classified	G89.2	1,188	164	13.805	1,260	142	11.270	2.535, -0.091-5.161	**1.631**, 1.290-2.062	< 0.0001
Acute pain, not elsewhere classified	G89.1	1,278	49	3.834	1,328	32	2.410	1.424, 0.087-2.762	**1.990**, 1.244-3.182	0.0035
Polyneuropathy, unspecified	G62.9	1,261	58	4.6	1,316	33	2.508	2.092, 0.66-3.524	**1.943**, 1.258-3.0	0.0023
Benign prostatic hyperplasia	N40	1,252	41	3.275	1,262	72	5.705	-2.43, -4.046- -0.815	**0.6**, 0.407-0.884	0.0090
Candidiasis	B37	1,219	73	5.989	1,311	40	3.051	2.937, 1.312-4.563	**2.377**, 1.592,3.55	< 0.0001
Dermatophytosis	B35	1,216	78	6.414	1,303	62	4.758	1.656, -0.142-3.454	**1.457**, 1.039-2.045	0.0283
Sepsis, unspecified organism	A41.9	1,270	51	4.016	1,326	32	2.413	1.602, 0.243-2.962	**1.713**, 1.097-2.676	0.0166
Nicotine dependence	F17	1,131	65	5.747	1,254	56	4.466	–	–	n.s.

The risk of individuals diagnosed with EBA cases to those without EBA controls to develop one of the listed diseases. Data shown displays results from measures of association excluding patients with outcome prior to the time window. For the Hazard ratio. Kaplan-Meier Analysis again excluding patients with outcome prior to the time window with Log-Rank Test was performed. N, number; n.s., not significant; n.a., not applicable; Diseases where presence of EBA increases the risk are highlighted in bold. Diseases where presence of EBA decreases the risk are highlighted in blue and bold. Diseases in which EBA does not impose a risk for subsequent disease development are displayed in gray letters. Please note that propensity score matching is re-run with each outcomes analysis so that the analysis uses the most current data available on the TriNetX network. Analysis was performed from October 1st to 3rd, 2022.

### Non-communicable inflammatory diseases are risk factors and sequelae of EBA

A total of 9 non-communicable inflammatory diseases (CID) were amongst the 55 diseases with a different prevalence contrasting EBA to no-EBA patients (**Tables 1**, **2** and **Figure 2**). Of these 9 CIDs, 6 were identified as risk factors for EBA. Considering that 23 diseases were identified as risk factors for EBA, CIDs are the most prevalent risk factor for subsequent EBA development. Lichen planus (HR 10.20, CI 2.39-43.50, p<0.0001), cutaneous lupus erythematosus (HR 26.96, CI 3.67-198.22, p<0.0001) and systemic lupus erythematosus (SLE, HR 3.10, CI 1.31-26.91, p=0.0087) were among the disease with the highest risk for future EBA development. Interestingly, “other chronic obstructive pulmonary disease” (COPD) was associated with a decreased risk of subsequent EBA development (HR 0.73, CI 0.55-0.98, p=0.0378).

Likewise, EBA was a risk factor for subsequent CID development. Of the 37 diseases for which EBA is a risk factor, 7 were CIDs. Again, lichen planus (HR 6.64, CI 1.74-25.28, p<0.0017), cutaneous lupus erythematosus (HR 15.55, CI 2.05-117.69, p=0.0004) and SLE (HR 5.94, CI 1.47-6.55, p=0.0018) were among the diseases manifesting following an EBA diagnosis. Albeit not a classical CID, pruritus was found to be both a risk factor (HR 3.51, CI 2.60-4.74, p<0.0001) and a sequel of EBA (HR 2.79, CI 2.01-3.88, p<0.0001).

Of note, inflammatory bowel disease, such as ulcerative colitis and Crohn’s disease, that had been previously assumed to be associated with EBA (12, 17–19) were not identified as risk factors or sequelae of EBA. Ulcerative colitis was not among the 55 diseases with a different prevalence when comparing EBA to no-EBA patients. Crohn’s disease was identified to have a different prevalence in EBA compared to no-EBA patients. However, in neither of the two, propensity matched, case-control studies, Crohn’s disease was identified as a risk factor or a sequela of EBA.

### Infectious diseases are risk factors and sequelae of EBA

Sepsis, dermatophytosis and candidiasis were among the 55 diseases with a different prevalence between EBA and no-EBA patients (**Tables 1**, **2**; **Figure 2**). Given that unspecific immunosuppression is the treatment of choice for EBA (12, 20–22), infectious diseases can be expected as sequelae of EBA. Indeed, sepsis, dermatophytosis and candidiasis were identified as sequelae of EBA. Interestingly, all were also risk factors for future EBA development: Sepsis (HR 1.94, CI 1.33-2.81, p=0.0004), dermatophytosis (HR 1.57, CI 1.14-2.15, p=0.0056) and candidiasis (HR 2.31, CI 1.64-3.27, p<0.0001).

### Malignant melanoma, non-melanoma skin cancer and neoplasms of the liver and intrahepatic bile ducts are risk factors and sequelae of EBA

Regarding malignancy, 3/55 identified diagnoses fell into this category; namely, non-melanoma skin cancer, melanoma and malignant neoplasm of the liver and intrahepatic bile ducts. Whilst non-melanoma skin cancer and malignant neoplasm of the liver and intrahepatic bile ducts were both risk factors and sequelae of EBA, melanoma was not associated with a higher risk to develop EBA, but developed more frequently after an EBA diagnosis (**Tables 1**, **2** and **Figure 2**).

### EBA has a considerable metabolic and cardiovascular disease risk

Metabolic and cardiovascular comorbidity significantly contributes to morbidity and mortality of CIDs (23–25). The potential metabolic and cardiovascular disease burden in EBA has, so far, not been addressed. Following the diagnosis of EBA, cerebral infarction (HR 2.04, CI 1.28-3.24, p=0.0004), deep vein thrombosis (HR 2.29, CI 11.30-4.04, p=0.0032), atherosclerosis (HR 1.44, CI 1.01-2.05, p=0.0407), heart failure (HR 1.58, CI 1.09-2.28, p=0.0147), hypertensive diseases (HR 1.29, CI 1.02-1.62, p=0.0350), type 2 diabetes mellitus (HR 1.42, CI 1.05-1.93, p=0.0245), and overweight & obesity (HR 1.36, CI 1.06-1.76, p=0.0156) developed more frequently than expected.

## Discussion

We here determined risk factors and sequelae of EBA using a large-scale database. This led to the identification of so far unrecognized disease trajectories, as well as to the refutation of long-held beliefs, such as an association of inflammatory bowel disease with EBA.

Because EBA is such a rare disease, we believe that implementation of preventive measures based on the identification of risk factors is, for the most part, impracticable. For example, the identified EBA risk factors dermatophytosis and candidiasis are common diseases (26). The effort to screen for EBA in these patient populations would not justify the potential benefit. We, however, see some exceptions. These are pruritus, conjunctival scars, and visual disturbances. If in any of these, a definite cause cannot be determined, EBA should be excluded as a potential differential diagnosis following current diagnostic recommendations (20, 27). By contrast, the identified metabolic and cardiovascular risk profile of EBA patients warrants clinical implementation. Compared to controls, EBA patients were more prone to develop hypertensive diseases, atherosclerosis, cerebral infarction, heart failure, deep vein thrombosis, type 2 diabetes mellitus and overweight & obesity – for some, the risk was increased over 2-fold (**Table 2** and **Figure 2**). This profile is in line with several other CIDs, for example SLE, rheumatoid arthritis and psoriasis (28–30). The finding of an increased frequency of deep vein thrombosis in EBA has also been made in bullous pemphigoid, where a prothrombotic state and an increased risk for venous thromboembolism had been noted (31, 32). Consequently, stringent control of known cardiovascular risk factors, and frequent screening for cardiovascular and metabolic disease should be implemented in the management of EBA patients.

We also found other CIDs to be risk factors and sequelae of EBA. Among these, lichen planus, SLE and cutaneous lupus were associated with the highest risk for both, future EBA development, and the probability of manifestation following an EBA diagnosis. Thus, it is tempting to speculate that EBA, lichen planus, SLE and cutaneous lupus share similar disease-driving pathomechanisms. These findings are also in line with previous clinical observations that noted a high prevalence of antinuclear autoantibodies in EBA patients (12, 33), and the co-occurrence of SLE with EBA, which coined the term bullous SLE (34–36).

Interestingly, inflammatory bowel diseases were not identified as risk factors or sequelae of EBA. An association of EBA with these had long been noted, with several subsequent publications supporting this assumption (5, 12, 17–19, 37). However, most of these observations were made before the establishment of the current diagnostic EBA criteria. Furthermore, these observations were based on single case reports, case report series and meta-analysis thereof. Considering the findings from our study, an association of EBA with inflammatory bowel disease seems rather unlikely.

Regarding malignancy, we think the increased risk of EBA patients to develop non-melanoma skin cancer and melanoma is due to regular and prolonged dermatological care. This is certainly not the case for malignant neoplasms of the liver and intrahepatic bile ducts, which were identified as a risk factor (HR 3.07, CI 1.74-5.31, p<0.0001) and a sequela of EBA (2.26, CI 1.42-3.60, p=0.0004). This necessitates regular screening for this malignancy, underscoring the need for a multidisciplinary care for EBA patients (38). The recently noted expression of COL7 in the liver (of mice) may explain this so far unnoticed association (39) – albeit this finding is not sustained by other reports (40). In the human protein atlas, *COL7A1* expression has, however, been reported for the gallbladder – and to a minor extent also in the liver. Thus, autoantibody-induced inflammation in the bile ducts may promote the emergence of malignant neoplasms of the liver and intrahepatic bile ducts.

Our study has several limitations to be acknowledged. First, patient electronic health record data may suffer from misdiagnosis and/or miscoding and do not encompass all possible confounding factors. In our dataset this is underscored by the diagnosis of benign prostatic hyperplasia, where close to 0.5% of individuals with this diagnosis were coded to be female (**Supplement Table 51**). Second, the TriNetX database provides access to medical data from individuals who had medical encounters with healthcare systems. Thus, our analysis does not include patients with low access to healthcare facilities. Third, in the risk factor analysis, the results must be interpreted with caution if the number of EBA cases is low.

In conclusion, the use of TriNetx allowed to define risk factors and sequalae of an orphan disease, here exemplified by EBA. The identified interactions have clinical implications for the management of EBA and point towards shared pathogenic pathways among different CIDs. We envision that the here described methodology will serve as a blueprint to identify risk factors and sequelae for numerus orphan diseases.

This work has only been possible because of Detlef Zillikens. Since 2004, he continuously and methodically developed a research infrastructure for pemphigus and pemphigoid diseases at the University of Lübeck. In doing so, he spread his enthusiasm for pemphigus and pemphigoid research to improve the diagnosis and treatment of patients suffering from these diseases to those around him. All authors decided to come to Lübeck because of Detlef Zillikens. We truly miss him as an inspiring and always motivating mentor and friend, and now seek to continue his mission in pemphigus and pemphigoid.

## Data availability statement

The original contributions presented in the study are included in the article/**Supplementary Material**. Further inquiries can be directed to the corresponding author.

## Author contributions

Conceptualization: KK, RL; Investigation: All authors; Illustrations: AV, KB; Project Administration: RL; Resources: RL; Writing - Original Draft Preparation: RL; Writing - Review and Editing: All authors. All authors contributed to the article and approved the submitted version
