# Supplementary material for: Risk factors and sequelae of epidermolysis bullosa acquisita: A propensity-matched global study in 1,344 patients
Source: Front Immunol. 2023 Jan 26;13:1103533. doi: 10.3389/fimmu.2022.1103533 (PMC9910332; doi:10.3389/fimmu.2022.1103533)
Supplement: Supplementary file 1 [file Table_1.docx]

**Supplement tables**

| **Characteristic** | **Overweight and obesity** | **Controls** |
| --- | --- | --- |
| Number of participants | 6,923,183 | 6,923,183 |
| Age in years (SD) | 46.3 ± 20.1 | 46.3 ± 20.1 |
| Female (%) | 60.421 | 60.421 |
| Not Hispanic or Latino (%) | 58.572 | 58.646* |
| White (%) | 61.907 | 61.907 |

**Supplement table 1.** Cohort description for ICD10:E66. Abbreviations: SD: standard deviation. *p<0.05

| **Characteristic** | **Type 2 diabetes mellitus** | **Controls** |
| --- | --- | --- |
| Number of participants | 5,637,777 | 5,637,777 |
| Age in years (SD) | 56.8 ± 16.8 | 57.2 ± 17.2* |
| Female (%) | 51.038 | 52.642* |
| Not Hispanic or Latino (%) | 57.633 | 61.215* |
| White (%) | 59.86 | 62.542* |

**Supplement table 2.** Cohort description for ICD10:E11. Abbreviations: SD: standard deviation. *p<0.05

| **Characteristic** | **Hypothyroidism, unspecified** | **Controls** |
| --- | --- | --- |
| Number of participants | 3,402,624 | 3,402,624 |
| Age in years (SD) | 57.7 ± 18.5 | 57.8 ± 18.5 |
| Female (%) | 74.413 | 74.528* |
| Not Hispanic or Latino (%) | 60.757 | 62.318* |
| White (%) | 73.151 | 73.452* |

**Supplement table 3.** Cohort description for ICD10:E03.9. Abbreviations: SD: standard deviation. *p<0.05

| **Characteristic** | **Malnutrition** | **Controls** |
| --- | --- | --- |
| Number of participants | 919,826 | 919,826 |
| Age in years (SD) | 58.7 ± 22.4 | 58.7 ± 22.4 |
| Female (%) | 49.693 | 49.693 |
| Not Hispanic or Latino (%) | 63.705 | 63.705 |
| White (%) | 63.881 | 63.881 |

**Supplement table 4.** Cohort description for ICD10: E40-E46. Abbreviations: SD: standard deviation.

| **Characteristic** | **Systemic lupus erythematosus** | **Controls** |
| --- | --- | --- |
| Number of participants | 231,354 | 231,354 |
| Age in years (SD) | 47.2 ± 17.6 | 47.2 ± 17.6 |
| Female (%) | 86.562 | 86.562 |
| Not Hispanic or Latino (%) | 62.409 | 62.409 |
| White (%) | 54.997 | 54.997 |

**Supplement table 5.** Cohort description for ICD10:M32. Abbreviations: SD: standard deviation. *p<0.05

| **Characteristic** | **Hypertensive diseases** | **Controls** |
| --- | --- | --- |
| Number of participants | 7,840,544 | 7,840,544 |
| Age in years (SD) | 53 ± 16.9 | 52.9 ± 17* |
| Female (%) | 54.25 | 53.917* |
| Not Hispanic or Latino (%) | n.d. | n.d. |
| White (%) | n.d. | n.d. |

**Supplement table 6.** Cohort description for ICD10: I10-I16. Abbreviations: SD: standard deviation, n.d.: not done, because the number of patients included does only allow to have 2 covariates for propensity matching. *p<0.05

| **Characteristic** | **Heart failure** | **Controls** |
| --- | --- | --- |
| Number of participants | 2,460,219 | 2,460,219 |
| Age in years (SD) | 65.3 ± 16.5 | 65.3 ± 16.4* |
| Female (%) | 48.521 | 47.247* |
| Not Hispanic or Latino (%) | 60.075 | 62.18* |
| White (%) | 62.74 | 62.671 |

**Supplement table 7.** Cohort description for ICD10:I509. Abbreviations: SD: standard deviation. *p<0.05

| **Characteristic** | **Atrial fibrillation and flutter** | **Controls** |
| --- | --- | --- |
| Number of participants | 2,440,273 | 2,440,273 |
| Age in years (SD) | 67.4 ± 14.1 | 67.4 ± 14.1 |
| Female (%) | 45.8 | 44.5* |
| Not Hispanic or Latino (%) | 60.1 | 63.3 |
| White (%) | 68.4 | 69.1* |

**Supplement table 8.** Cohort description for ICD10:I48. Abbreviations: SD: standard deviation. *p<0.05

| **Characteristic** | **Nonrheumatic mitral valve disorders** | **Controls** |
| --- | --- | --- |
| Number of participants | 1,549,211 | 1,549,211 |
| Age in years (SD) | 62.8 ± 18 | 62.8 ± 18 |
| Female (%) | 56.008 | 56.008 |
| Not Hispanic or Latino (%) | 68.882 | 68.882 |
| White (%) | 68.433 | 68.433 |

**Supplement table 9.** Cohort description for ICD10:I34. Abbreviations: SD: standard deviation.

| **Characteristic** | **Atherosclerosis** | **Controls** |
| --- | --- | --- |
| Number of participants | 1,246,369 | 1,246,369 |
| Age in years (SD) | 68.6 ± 13 | 68.6 ± 13 |
| Female (%) | 47.588 | 47.588 |
| Not Hispanic or Latino (%) | 59.071 | 59.071 |
| White (%) | 64.699 | 64.699 |

**Supplement table 10.** Cohort description for ICD10:I70. Abbreviations: SD: standard deviation.

| **Characteristic** | **Acute embolism and thrombosis of deep veins of lower extremity** | **Controls** |
| --- | --- | --- |
| Number of participants | 845,473 | 845,473 |
| Age in years (SD) | 58.9 ± 18.5 | 58.9 ± 18.5 |
| Female (%) | 51.885 | 51.885 |
| Not Hispanic or Latino (%) | 72.757 | 72.757 |
| White (%) | 70.797 | 70.797 |

**Supplement table 11.** Cohort description for ICD10:I82.4. Abbreviations: SD: standard deviation.

| **Characteristic** | **Hypotension** | **Controls** |
| --- | --- | --- |
| Number of participants | 1,837,200 | 1,837,200 |
| Age in years (SD) | 61 ± 20.1 | 61 ± 20.1 |
| Female (%) | 51.271 | 51.22 |
| Not Hispanic or Latino (%) | 64.85 | 64.932 |
| White (%) | 67.513 | 67.367* |

**Supplement table 12.** Cohort description for ICD10:I95. Abbreviations: SD: standard deviation. *p<0.05

| **Characteristic** | **Cerebral infarction** | **Controls** |
| --- | --- | --- |
| Number of participants | 1,567,640 | 1,567,640 |
| Age in years (SD) | 63.2 ± 19 | 63.2 ± 18.9 |
| Female (%) | 51.194 | 51.194* |
| Not Hispanic or Latino (%) | 63.757 | 64.425* |
| White (%) | 61.98 | 62.215* |

**Supplement table 13.** Cohort description for ICD10:I63. Abbreviations: SD: standard deviation. *p<0.05

| **Characteristic** | **Diverticular disease of intestine** | **Controls** |
| --- | --- | --- |
| Number of participants | 2,337,172 | 2,337,172 |
| Age in years (SD) | 63.7 ± 13.3 | 63.7 ± 13.3 |
| Female (%) | 53.495 | 53.362* |
| Not Hispanic or Latino (%) | 58.056 | 58.147* |
| White (%) | 69.761 | 69.761 |

**Supplement table 14.** Cohort description for ICD10:K57. Abbreviations: SD: standard deviation. *p<0.05

| **Characteristic** | **Gastro-esophageal reflux disease** | **Controls** |
| --- | --- | --- |
| Number of participants | 6,973,715 | 6,973,715 |
| Age in years (SD) | 50.3 ± 21.8 | 50.3 ± 21.9 |
| Female (%) | 57.74 | 57.502* |
| Not Hispanic or Latino (%) | 62.533 | 63.998* |
| White (%) | 67.762 | 67.647* |

**Supplement table 15.** Cohort description for ICD10:K21. Abbreviations: SD: standard deviation. *p<0.05

| **Characteristic** | **Ulcer of esophagus** | **Controls** |
| --- | --- | --- |
| Number of participants | 127,407 | 127,407 |
| Age in years (SD) | 59.1 ± 18.2 | 59.1 ± 18.2 |
| Female (%) | 44.31 | 44.31 |
| Not Hispanic or Latino (%) | 56.717 | 56.717 |
| White (%) | 67.685 | 67.685 |

**Supplement table 16.** Cohort description for ICD10:K22.1. Abbreviations: SD: standard deviation.

| **Characteristic** | **Gastritis and duodenitis** | **Controls** |
| --- | --- | --- |
| Number of participants | 1,869,594 | 1,869,594 |
| Age in years (SD) | 49.4 ± 21.7 | 49.4 ± 21.7 |
| Female (%) | 58.174 | 58.174 |
| Not Hispanic or Latino (%) | 50.456 | 50.456 |
| White (%) | 57.499 | 57.499 |

**Supplement table 17.** Cohort description for ICD10:K29. Abbreviations: SD: standard deviation.

| **Characteristic** | **Stomatitis and related lesions** | **Controls** |
| --- | --- | --- |
| Number of participants | 624,475 | 624,475 |
| Age in years (SD) | 39.2 ± 24.4 | 39.2 ± 24.4 |
| Female (%) | 54.353 | 54.353 |
| Not Hispanic or Latino (%) | 59.336 | 59.336 |
| White (%) | 59.704 | 59.704 |

**Supplement table 18.** Cohort description for ICD10:K12. Abbreviations: SD: standard deviation.

| **Characteristic** | **Diaphragmatic hernia** | **Controls** |
| --- | --- | --- |
| Number of participants | 1,329,647 | 1,329,647 |
| Age in years (SD) | 61.8 ± 16.6 | 61.8 ± 16.6 |
| Female (%) | 61.151 | 61.151 |
| Not Hispanic or Latino (%) | 56.178 | 56.178 |
| White (%) | 67.745 | 67.745 |

**Supplement table 19.** Cohort description for ICD10:K44. Abbreviations: SD: standard deviation.

| **Characteristic** | **Crohn's disease** | **Controls** |
| --- | --- | --- |
| Number of participants | 294,567 | 294,567 |
| Age in years (SD) | 43.2 ± 20.1 | 43.2 ± 20.1 |
| Female (%) | 55.561 | 55.561 |
| Not Hispanic or Latino (%) | 63.218 | 63.218 |
| White (%) | 69.877 | 69.877 |

**Supplement table 20.** Cohort description for ICD10:K50. Abbreviations: SD: standard deviation.

| **Characteristic** | **Other chronic obstructive pulmonary disease** | **Controls** |
| --- | --- | --- |
| Number of participants | 2,317,998 | 2,317,998 |
| Age in years (SD) | 65.4 ± 14.3 | 65.5 ± 14.4* |
| Female (%) | 50.952 | 51.565* |
| Not Hispanic or Latino (%) | 56.777 | 58.806* |
| White (%) | 68.741 | 69.002* |

**Supplement table 21.** Cohort description for ICD10:J44. Abbreviations: SD: standard deviation. *p<0.05

| **Characteristic** | **Sleep disorders** | **Controls** |
| --- | --- | --- |
| Number of participants | 5,970,833 | 5,970,833 |
| Age in years (SD) | 48.2 ± 22.1 | 48.3 ± 22.2 |
| Female (%) | 51.012 | 51.209* |
| Not Hispanic or Latino (%) | 65.507 | 65.523 |
| White (%) | 68.502 | 68.508 |

**Supplement table 22.** Cohort description for ICD10:G47. Abbreviations: SD: standard deviation. *p<0.05

| **Characteristic** | **Acute kidney failure and chronic kidney disease** | **Controls** |
| --- | --- | --- |
| Number of participants | 3,557,385 | 3,557,385 |
| Age in years (SD) | 60.9 ± 17.8 | 60.9 ± 17.8* |
| Female (%) | 47.947 | 47.058* |
| Not Hispanic or Latino (%) | 59.201 | 61.517* |
| White (%) | 61.559 | 61.528 |

**Supplement table 23.** Cohort description for ICD10:N17-N19. Abbreviations: SD: standard deviation. *p<0.05

| **Characteristic** | **Malignant melanoma of skin** | **Controls** |
| --- | --- | --- |
| Number of participants | 282,476 | 282,476 |
| Age in years (SD) | 61.2 ± 16.6 | 61.2 ± 16.6 |
| Female (%) | 48.073 | 48.073 |
| Not Hispanic or Latino (%) | 66.816 | 66.816 |
| White (%) | 78.411 | 78.411 |

**Supplement table 24.** Cohort description for ICD10:C43. Abbreviations: SD: standard deviation.

| **Characteristic** | **Malignant neoplasm of liver and intrahepatic bile ducts** | **Controls** |
| --- | --- | --- |
| Number of participants | 594,491 | 594,491 |
| Age in years (SD) | 56.2 ± 19.6 | 56.2 ± 19.6 |
| Female (%) | 49.948 | 49.948 |
| Not Hispanic or Latino (%) | 79.477 | 79.477 |
| White (%) | 75.17 | 75.17 |

**Supplement table 25.** Cohort description for ICD10:C22. Abbreviations: SD: standard deviation.

| **Characteristic** | **Melanocytic nevi** | **Controls** |
| --- | --- | --- |
| Number of participants | 1,370,800 | 1,370,800 |
| Age in years (SD) | 46.5 ± 21 | 46.5 ± 21 |
| Female (%) | 59.641 | 59.641 |
| Not Hispanic or Latino (%) | 68.404 | 68.404 |
| White (%) | 78.928 | 78.928 |

**Supplement table 26.** Cohort description for ICD10:D22. Abbreviations: SD: standard deviation.

| **Characteristic** | **Other and unspecified malignant neoplasm of skin** | **Controls** |
| --- | --- | --- |
| Number of participants | 1,080,690 | 1,080,690 |
| Age in years (SD) | 66.8 ± 14.7 | 66.8 ± 14.7 |
| Female (%) | 45.922 | 45.922 |
| Not Hispanic or Latino (%) | 62.694 | 62.694 |
| White (%) | 75.13 | 75.13 |

**Supplement table 27.** Cohort description for ICD10:C44. Abbreviations: SD: standard deviation.

| **Characteristic** | **Anemia, unspecified** | **Controls** |
| --- | --- | --- |
| Number of participants | 4,097,593 | 4,097,593 |
| Age in years (SD) | 53.4 ± 22.9 | 53.2 ± 22.7* |
| Female (%) | 60.651 | 62.2* |
| Not Hispanic or Latino (%) | 60.476 | 61.179* |
| White (%) | 58.919 | 58.513* |

**Supplement table 28.** Cohort description for ICD10:D64.9. Abbreviations: SD: standard deviation. *p<0.05

| **Characteristic** | **Elevated white blood cell count** | **Controls** |
| --- | --- | --- |
| Number of participants | 1,554,638 | 1,554,638 |
| Age in years (SD) | 53.4 ± 21.9 | 53.4 ± 21.9 |
| Female (%) | 51.828 | 51.828 |
| Not Hispanic or Latino (%) | 67.851 | 67.851 |
| White (%) | 70.287 | 70.287 |

**Supplement table 29.** Cohort description for ICD10:D72.82. Abbreviations: SD: standard deviation.

| **Characteristic** | **Purpura and other hemorrhagic conditions** | **Controls** |
| --- | --- | --- |
| Number of participants | 1,325,334 | 1,325,334 |
| Age in years (SD) | 55.1 ± 23.0 | 55.1 ± 23.0 |
| Female (%) | 46.5 | 46.5 |
| Not Hispanic or Latino (%) | 62.1 | 62.1 |
| White (%) | 66.1 | 66.1 |

**Supplement table 30.** Cohort description for ICD10:D69. Abbreviations: SD: standard deviation. *p<0.05

| **Characteristic** | **Iron deficiency anemia** | **Controls** |
| --- | --- | --- |
| Number of participants | 2,055,380 | 2,055,380 |
| Age in years (SD) | 53.1 ± 22.8 | 53.1 ± 22.8 |
| Female (%) | 67.561 | 67.561 |
| Not Hispanic or Latino (%) | 57.648 | 57.767* |
| White (%) | 55.039 | 55.039 |

**Supplement table 31.** Cohort description for ICD10:D50. Abbreviations: SD: standard deviation. *p<0.05

| **Characteristic** | **Age-related cataract** | **Controls** |
| --- | --- | --- |
| Number of participants | 1,663,918 | 1,663,918 |
| Age in years (SD) | 66.1 ± 10.8 | 66.1 ± 10.8 |
| Female (%) | 56.656 | 56.656 |
| Not Hispanic or Latino (%) | 57.271 | 57.271 |
| White (%) | 60.352 | 60.352 |

**Supplement table 32.** Cohort description for ICD10:H25. Abbreviations: SD: standard deviation.

| **Characteristic** | **Other cataract** | **Controls** |
| --- | --- | --- |
| Number of participants | 1,223,336 | 1,223,336 |
| Age in years (SD) | 67.8 ± 14.9 | 67.9 ± 15* |
| Female (%) | 57.82 | 57.015* |
| Not Hispanic or Latino (%) | 57.426 | 58.321* |
| White (%) | 59.224 | 60.055* |

**Supplement table 33.** Cohort description for ICD10:H26. Abbreviations: SD: standard deviation. *p<0.05

| **Characteristic** | **Visual disturbances and blindness** | **Controls** |
| --- | --- | --- |
| Number of participants | 3,457,301 | 3,457,301 |
| Age in years (SD) | 44.9 ± 24.6 | 44.9 ± 24.6 |
| Female (%) | 56.494 | 56.494 |
| Not Hispanic or Latino (%) | 65.237 | 65.237 |
| White (%) | 63.661 | 63.661 |

**Supplement table 34.** Cohort description for ICD10:H53-H54. Abbreviations: SD: standard deviation.

| **Characteristic** | **Glaucoma** | **Controls** |
| --- | --- | --- |
| Number of participants | 1,222,017 | 1,222,017 |
| Age in years (SD) | 63.3 ± 17.6 | 63.3 ± 17.6 |
| Female (%) | 56.026 | 56.026 |
| Not Hispanic or Latino (%) | 59.921 | 59.921 |
| White (%) | 55.837 | 55.837 |

**Supplement table 35.** Cohort description for ICD10:H40-H42. Abbreviations: SD: standard deviation.

| **Characteristic** | **Dry eye syndrome** | **Controls** |
| --- | --- | --- |
| Number of participants | 908,711 | 908,711 |
| Age in years (SD) | 58.9 ± 17.6 | 58.9 ± 17.6 |
| Female (%) | 69.289 | 69.289 |
| Not Hispanic or Latino (%) | 62.849 | 62.849 |
| White (%) | 61.461 | 61.461 |

**Supplement table 36.** Cohort description for ICD10:H04.12. Abbreviations: SD: standard deviation.

| **Characteristic** | **Presbyopia** | **Controls** |
| --- | --- | --- |
| Number of participants | 878,577 | 878,577 |
| Age in years (SD) | 59.7 ± 12.7 | 59.7 ± 12.7 |
| Female (%) | 58.692 | 58.692 |
| Not Hispanic or Latino (%) | 63.962 | 63.962 |
| White (%) | 65.299 | 65.299 |

**Supplement table 37.** Cohort description for ICD10:H52.4. Abbreviations: SD: standard deviation.

| **Characteristic** | **Conjunctival scars** | **Controls** |
| --- | --- | --- |
| Number of participants | 11,573 | 11,573 |
| Age in years (SD) | 51.6 ± 23.8 | 51.6 ± 23.8 |
| Female (%) | 49.382 | 49.382 |
| Not Hispanic or Latino (%) | 62.136 | 62.136 |
| White (%) | 61.505 | 61.505 |

**Supplement table 38.** Cohort description for ICD10:H11.2. Abbreviations: SD: standard deviation.

| **Characteristic** | **Keratitis** | **Controls** |
| --- | --- | --- |
| Number of participants | 362,113 | 362,113 |
| Age in years (SD) | 51.2 ± 21.7 | 51.2 ± 21.7 |
| Female (%) | 59.518 | 59.518 |
| Not Hispanic or Latino (%) | 53.4 | 53.4 |
| White (%) | 58.731 | 58.731 |

**Supplement table 39.** Cohort description for ICD10:H16. Abbreviations: SD: standard deviation.

| **Characteristic** | **Disorders of choroid and retina** | **Controls** |
| --- | --- | --- |
| Number of participants | 1,476,257 | 1,476,257 |
| Age in years (SD) | 59.7 ± 22.8 | 59.7 ± 22.8 |
| Female (%) | 54.774 | 54.774 |
| Not Hispanic or Latino (%) | 58.935 | 58.935 |
| White (%) | 61.578 | 61.578 |

**Supplement table 40.** Cohort description for ICD10:H30-H36. Abbreviations: SD: standard deviation.

| **Characteristic** | **Lupus erythematosus** | **Controls** |
| --- | --- | --- |
| Number of participants | 92,904 | 92,904 |
| Age in years (SD) | 47.5 ± 18.3 | 47.5 ± 18.3 |
| Female (%) | 82.624 | 82.624 |
| Not Hispanic or Latino (%) | 69.396 | 69.396 |
| White (%) | 58.104 | 58.104 |

**Supplement table 41.** Cohort description for ICD10:L93. Abbreviations: SD: standard deviation.

| **Characteristic** | **Pressure ulcer** | **Controls** |
| --- | --- | --- |
| Number of participants | 520,287 | 520,287 |
| Age in years (SD) | 66.2 ± 20.6 | 66.2 ± 20.6 |
| Female (%) | 49.327 | 49.052* |
| Not Hispanic or Latino (%) | 56.441 | 56.441 |
| White (%) | 62.077 | 62.077 |

**Supplement table 42.** Cohort description for ICD10:L89. Abbreviations: SD: standard deviation. *p<0.05

| **Characteristic** | **Pruritus** | **Controls** |
| --- | --- | --- |
| Number of participants | 1,719,784 | 1,719,784 |
| Age in years (SD) | 43.3 ± 23.8 | 43.3 ± 23.8 |
| Female (%) | 65.87 | 65.87 |
| Not Hispanic or Latino (%) | 69.49 | 69.49 |
| White (%) | 64.204 | 64.204 |

**Supplement table 43.** Cohort description for ICD10:L29. Abbreviations: SD: standard deviation.

| **Characteristic** | **Seborrheic dermatitis** | **Controls** |
| --- | --- | --- |
| Number of participants | 667,688 | 667,688 |
| Age in years (SD) | 36.1 ± 27.8 | 36.1 ± 27.8 |
| Female (%) | 52.44 | 52.44 |
| Not Hispanic or Latino (%) | 67.433 | 67.433 |
| White (%) | 60.558 | 60.558 |

**Supplement table 44.** Cohort description for ICD10:L21. Abbreviations: SD: standard deviation.

| **Characteristic** | **Lichen simplex chronicus and prurigo** | **Controls** |
| --- | --- | --- |
| Number of participants | 258,035 | 258,035 |
| Age in years (SD) | 48.9 ± 23 | 48.9 ± 23 |
| Female (%) | 60.069 | 60.069 |
| Not Hispanic or Latino (%) | 66.92 | 66.92 |
| White (%) | 60.767 | 60.767 |

**Supplement table 45.** Cohort description for ICD10:L28. Abbreviations: SD: standard deviation.

| **Characteristic** | **Urticaria** | **Controls** |
| --- | --- | --- |
| Number of participants | 1,072,297 | 1,072,297 |
| Age in years (SD) | 31.9 ± 24.2 | 31.9 ± 24.2 |
| Female (%) | 63.122 | 63.122 |
| Not Hispanic or Latino (%) | 62.71 | 62.71 |
| White (%) | 61.504 | 61.504 |

**Supplement table 46.** Cohort description for ICD10:L50.0. Abbreviations: SD: standard deviation.

| **Characteristic** | **Lichen planus** | **Controls** |
| --- | --- | --- |
| Number of participants | 74,610 | 74,610 |
| Age in years (SD) | 57.4 ± 16.9 | 57.4 ± 16.9 |
| Female (%) | 68.567 | 68.567 |
| Not Hispanic or Latino (%) | 68.219 | 68.219 |
| White (%) | 65.495 | 65.495 |

**Supplement table 47.** Cohort description for ICD10:L43. Abbreviations: SD: standard deviation.

| **Characteristic** | **Chronic pain, not elsewhere classified** | **Controls** |
| --- | --- | --- |
| Number of participants | 4,199,744 | 4,199,744 |
| Age in years (SD) | 52.5 ± 18.5 | 52.5 ± 18.5 |
| Female (%) | 58.609 | 58.609 |
| Not Hispanic or Latino (%) | 64.708 | 64.708 |
| White (%) | 70.039 | 70.039 |

**Supplement table 48.** Cohort description for ICD10:G89.2. Abbreviations: SD: standard deviation.

| **Characteristic** | **Acute pain, not elsewhere classified** | **Controls** |
| --- | --- | --- |
| Number of participants | 1,261,353 | 1,261,353 |
| Age in years (SD) | 49.1 ± 21 | 49.1 ± 21 |
| Female (%) | 53.408 | 53.408 |
| Not Hispanic or Latino (%) | 70.174 | 70.174 |
| White (%) | 69.37 | 69.37 |

**Supplement table 49.** Cohort description for ICD10:G89.1. Abbreviations: SD: standard deviation.

| **Characteristic** | **Polyneuropathy, unspecified** | **Controls** |
| --- | --- | --- |
| Number of participants | 1,013,132 | 1,013,132 |
| Age in years (SD) | 61 ± 15.9 | 61 ± 15.9 |
| Female (%) | 53.876 | 53.876 |
| Not Hispanic or Latino (%) | 53.876 | 53.876 |
| White (%) | 72.67 | 72.67 |

**Supplement table 50.** Cohort description for ICD10:G65.9. Abbreviations: SD: standard deviation.

| **Characteristic** | **Benign prostatic hyperplasia** | **Controls** |
| --- | --- | --- |
| Number of participants | 1,593,589 | 1,593,589 |
| Age in years (SD) | 65.8 ± 11.1 | 65.8 ± 11.1 |
| Female (%) | 0.467* | 0.467* |
| Not Hispanic or Latino (%) | 58.848 | 58.848** |
| White (%) | 67.104 | 67.104** |

**Supplement table 51.** Cohort description for ICD10:N40. Abbreviations: SD: standard deviation. *reflecting coding errors, **p<0.05

| **Characteristic** | **Candidiasis** | **Controls** |
| --- | --- | --- |
| Number of participants | 1,933,740 | 1,933,740 |
| Age in years (SD) | 38.7 ± 25.7 | 38.7 ± 25.7 |
| Female (%) | 75.49 | 75.49 |
| Not Hispanic or Latino (%) | 61.245 | 61.245 |
| White (%) | 58.764 | 58.764 |

**Supplement table 52.** Cohort description for ICD10:B37. Abbreviations: SD: standard deviation.

| **Characteristic** | **Dermatophytosis** | **Controls** |
| --- | --- | --- |
| Number of participants | 1,677,004 | 1,677,004 |
| Age in years (SD) | 47.2 ± 24.8 | 47.2 ± 24.8 |
| Female (%) | 48.506 | 48.506 |
| Not Hispanic or Latino (%) | 60.916 | 60.916 |
| White (%) | 59.406 | 59.406 |

**Supplement table 53.** Cohort description for ICD10:B35. Abbreviations: SD: standard deviation.

| **Characteristic** | **Sepsis, unspecified organism** | **Controls** |
| --- | --- | --- |
| Number of participants | 1,337,037 | 1,337,037 |
| Age in years (SD) | 59.7 ± 21.5 | 59.7 ± 21.5 |
| Female (%) | 48.069 | 48.069 |
| Not Hispanic or Latino (%) | 56.945 | 56.945 |
| White (%) | 62.601 | 62.533 |

**Supplement table 54.** Cohort description for ICD10:A41.9. Abbreviations: SD: standard deviation.

| **Characteristic** | **Nicotine dependence** | **Controls** |
| --- | --- | --- |
| Number of participants | 5,515,840 | 5,515,840 |
| Age in years (SD) | 45.8 ± 16.8 | 45.8 ± 16.8* |
| Female (%) | 46.751 | 46.751 |
| Not Hispanic or Latino (%) | 57.757 | 57.996* |
| White (%) | 64.847 | 64.847 |

**Supplement table 55.** Cohort description for ICD10:F17. Abbreviations: SD: standard deviation. *p<0.05

| **Characteristic** | **EBA** | **Controls** |
| --- | --- | --- |
| Number of participants | 1,344 | 1,344 |
| Age in years (SD) | 57 ± 18.9 | 56.9 ± 18.9 |
| Female (%) | 52.307 | 52.307 |
| Not Hispanic or Latino (%) | 50.670 | 50.372 |
| White (%) | 68.676 | 68.378 |

**Supplement table 56.** Cohort description, sequelae of EBA. Abbreviations: EBA: Epidermolysis bullosa acquisita, SD: standard deviation.
